# Targeting Glia Cells: Novel Perspectives for the Treatment of Neuropsychiatric Diseases

**DOI:** 10.2174/1570159X11311020004

**Published:** 2013-03

**Authors:** B Di Benedetto, R Rupprecht

**Affiliations:** 1 Max Planck Institute of Psychiatry, Munich, Germany; 2 Department of Psychiatry and Psychotherapy, Regensburg University, Germany

**Keywords:** Astrocytes, microglia, neuropsychiatric disorders, oligodendrocytes, pharmacotherapy

## Abstract

Neuropsychiatric disorders are devastating mental illnesses with a high economic burden. The additional morbidity associated with social issues that arises along with the course of these diseases increases the need for a clear understanding of their etiopathogenesis to allow an implementation of novel pharmacological strategies. Yet a poor knowledge about interactions occurring at the glia-neuron interface in health and disease still hampers innovative discoveries, despite the fact that glia cells have been long described to actively participate in the regulation of brain circuits.

The purpose of this review was to collect the scattered literature on the involvement of glia cells in neuropsychiatric disorders and to describe how also these cells besides neurons might be responsive to current pharmacological interventions. We hope thereby to offer alternative approaches for investigations that may open avenues to search for new potential targets for drug discovery.

## 1 INTRODUCTION

Neuropsychiatric disorders represent a high economical burden for many countries. Nonetheless, a clear understanding of their etiopathogenesis still remains elusive. Several methodological approaches have been further developed with the hope to get better insights into molecular and environmental factors that might lead to the development of such disorders. Pharmacogenetics and linkage disequilibrium studies offered unbiased, hypothesis-free approaches to allow the identification of putative candidate genes associated with several CNS diseases. Moreover, functional imaging has greatly improved in its sensitivity to offer a screening opportunity for a categorization of specific disease subtypes, independent from questionnaires which are mostly related to subjective interpretations of patients´s responses. Nevertheless, though many of these studies shed lights on new mechanisms related to development or progress of neuropsychiatric diseases, we are still far from a complete understanding of their pathophysiology. In fact, though the identification of some pathogenic mechanisms and modes of action of commonly used pharmacological compounds led to the development of better medicaments regarding side effects profile and tolerability, still the unsatisfactory efficacy of many of them and the insufficient response rates represent undoubted critical problems in the clinical course to remission. Indeed, for most of these diseases, first line medications are still based on old-developed drugs, such as for example antidepressants or antipsychotics, which are based on their modes of action discovered in the 60´s. Therefore, it is of primary importance to introduce new approaches to look at CNS disorders that might help to put forward both basic and clinic neuropsychiatric research.

To try to maintain what was called “the promise of personalized medicine” [1], research in the last three-four decades realized that the high heterogeneity of cell types populating the brain might have offered more than only a simple field of observation, but indeed it may have represented an invaluable source of new information on brain´s functions. Among the cell types that got increased attention in these years, glia cells have appeared in all their multifaceted spectrum of activities thank to the seminal work of several scientists. Nevertheless, many reports are still found scattered in the literature and are not much useful so “uncollected”. Therefore, with this review we wanted to collect together the abundant information available on these fascinating cell types that serve not only an invaluable supportive role for several brain functions, but which can also become a priceless tool to specifically refine pharmacological treatments, once our knowledge about their functions (or dysfunctions) becomes more available.

For a thorough description of the origin of the different glia subtypes, we better remit the reader to more specialized reviews that discuss this topic [2, 3]. Moreover, several excellent reviews have been produced that discuss the role of one or the other glia cell type in neuropsychiatric diseases. Most of them focused on astrocytes, as these were first described to play alternative roles in brain functions, such as in the modulation of synaptic plasticity at the so-called tripartite synapse [4, 5]. Few also explored the possible involvement of other glia cell populations in some disorders [6-8].

Therefore, in this review we will only briefly summarize recent advances on the mostly known features of glia cells in the postnatal CNS with the specific aim of offering a general introduction on their heterogeneity and numerous functions. Then, we will propose how glia cells might be seen as a common “central thread” in the pathogenesis of different psychiatric diseases. Finally, we will more thoroughly discuss a complementary “gliocentric” approach to improve our understanding of glia cells responsiveness to the major therapeutic pharmacological interventions used as first line medication in neuropsychiatric disorders. With this review, we aim to make a first glance at alternative perspectives when looking at modes of action of commonly used pharmacological compounds that might be of encouragement to approach neuropsychiatric disorders and pharmacological treatments with “other eyes”. Novel insights might indeed be necessary to gain valuable information for the identification of new targets amenable for drug discovery.

## 2 GLIA CELLS: A BRIEF EXCURSUS ON THEIR IDENTITY AND FUNCTIONS IN THE CENTRAL NERVOUS SYSTEM (CNS)

Glia cells represent the most diffused cell type in the central nervous system (CNS), with regional specificities regarding their numbers and ratio between neuronal and glial populations [9]. Nevertheless, for many years they have been only marginally considered to actively participate to neuronal network activity. Instead, their passive supportive role to neuronal functions has been mostly highlighted. Only the last ten-fifteen years have seen a growing body of literature that reported on new aspects of glia cells´s functions. 

A major subdivision of glia cells into two main groups, the macroglia and the microglia cells, was already proposed a century ago, based on their gross morphological differences. Further immunohistochemical and dye-labelling studies refined then this classification: macroglia cells can be further subdivided into astrocytes and oligodendrocytes, while microglia cells represent the immunosystem of the brain and execute functions more similarly related to those of macrophages in the bloodstream [10]. In Fig. (**1**) these major cell types are depicted in an arrangement that should represent their mostly known functions: *i*) astrocytes, *ii*) oligodendrocytes and *iii*) microglia. Each of them plays various roles, some of which may be interchangeable, while others appear to be very unique of each cell type. A common characteristic that has been often described in psychiatric patients, which derived from the evolution of methods such as functional Magnetic Resonance Imaging (fMRI), is that some of the brain regions involved in the processing of emotional stimuli are either enlarged or reduced in their volumes [11-13]. Of course, the question immediately arose whether these morphological changes might represent a pre-existing condition prior to the onset of a disease or develop as a consequence of its progression. This question may, unfortunately, hardly be answered due to the fact that when human postmortem tissues from affected patients become available to histological examination or a patient is subjected to fMRI, a diagnosis of disease is already present and it is not any longer feasible to know whether the morphological changes were already present before the onset of the disease, or came afterwards. Nonetheless, this intriguing observation has fostered several studies aimed at characterizing the neurobiological substrates of such morphologic changes. 

Among the main findings, the reduced brain areas have been associated with either reduced complexity of neuronal circuits or with overall reductions in cell numbers.

### 2.1 Astrocytes

The first subdivision of astrocytes into two major categories, fibrous and protoplasmic, was made already after the observation of their differential morphologies and their location either in the white or in the gray matter, respectively [14]. Fibrous astrocytes of the white matter show a more typical “star-like” appearance, with regular cylindrical processes that may interact with oligodendrocytes and show a high content of the glial fibrillary acidic protein GFAP. Protoplasmic astrocytes, instead, have a less regular morphology, extend more processes of distinct thicknesses that protrude to get in contact with several other brain structures and contained much less of the intermediate filament marker GFAP [15]. For the interactions that astrocytes can form, they may be considered as “bridging elements” of the brain that produce modulating signals and functionally connect together structures otherwise lacking any kind of communication. For example, their contribution in coupling the vasculature system with neuronal circuits in the so-called “neurovascular unit” has been strongly highlighted by recent research work [16, 17]. Through their end-feet that contact blood vessels, astrocytes can regulate transport of substances in/out of the brain, to ensure a proper homeostasis of ions and nutrients, but also excretion of catabolites that may exert toxic effects [18]. They can additionally modulate cerebral blood flow through an orchestrated release of several molecular messengers [16]. Furthermore, they can influence the maintenance of the blood brain barrier (BBB), whose proper functionality has fundamental consequences on the entire brain homeostasis [19]. Moreover, their role at the “tripartite synapse” has been extensively studied for the active regulation of glutamatergic signaling [20], lactate transport as energy supply for active neurons [17] or regulation of neurotransmitters´s diffusion [21]. In addition to these roles in the adult brain, the supportive influence of this cell type on the modulation of induction and stabilization of functional synaptic contacts has been also a very recent fruitful field of research [22-24]. In these ways, astrocytes can control many important activities of the brain and represent a unique system for the modulation of neuronal activity [21, 25]. Thus, they may also affect many behaviors that are dependent on a balanced neuronal network activity. 

Two further classes of glia cells do exist, that can be found only in restricted areas of the CNS and show a “radial” phenotype: Bergmann glia cells are the astrocytes that populate the cerebellum [26], while Muller glia cells are those in the retina [27]. Though they are different in their morphology in respect to other protoplasmic astrocytes, they share with them a polarized orientation of their processes that get in contact with synapses to regulate neurotransmission [28].

### 2.2 Oligodendrocytes

The second population of glia cells that we would like to discuss in this review is represented by oligodendrocytes. Their mostly known feature consists in the formation of myelin sheaths around axons to facilitate long range transmission of action potentials in the CNS. In the PNS, instead, Schwann cells are the glia cells that myelinate axons [29]. Mature oligodendrocytes in the CNS derive from oligodendrocyte progenitor cells (OPC) that migrate into the CNS during early development and progressively differentiate into mature oligodendrocytes in parallel with the maturation of the axons that they myelinate [30]. Though considered for long time to be fundamental for myelination, increasing literature is now proposing that oligodendrocytes may also actively shape neuronal activity. More specifically, OPC have been found to play important roles in neuronal transmission. Recognizable by the expression of the peculiar membrane proteoglycan NG2, this cell type has been described to participate in formation of long-term potentiation [31]. Previous work, indeed, had conclusively demonstrated the presence of glutamatergic synapses on NG2 positive cells and that glutamate is actively released from these synapses [32]. More recently, a new report confirmed the existence of glutamatergic synaptic inputs onto NG2 positive cells and showed how sensory experience regulates their proliferation, thereby controlling their distribution in the barrel cortex during development [33]. 

### 2.3 Microglia 

Microglia cells represent the resident immune cells of the CNS, with an important function in cleaning cell debris through phagocytosis mechanisms [34]. Only in recent years, though, the discovery that microglia cells contribute during development to the refinement of neuronal circuits actively participating in synaptic pruning has opened the avenue for a new research field that focuses on this cell type. In particular, work of Stevens and colleagues [35] has highlighted the role of the complement cascade in the selective elimination of inappropriate synaptic connections *via *expression of the complement proteins C1q and C3 that, when activated, get recognized by C3 receptors located on microglia cells. Thereby, they suggested a potential role of microglia cells in neuropsychiatric diseases through either an excessive or insufficient stripping of synaptic contacts. Further work on this cell type has contributed to clarify how microglia cells are supporting brain functions not only as surveyors in the brain, but also as key modulators to shape neuronal circuits during early postnatal development [36, 37]. 

In addition, microglia cells have been shown to sense neuronal activity and be crucial for long-term potentiation, a cellular correlate of learning and memory formation [38].

## 3 GLIA CELLS AND NEUROPSYCHIATRIC DISORDERS: EVIDENCES THAT GLIA CELLS MAY CONTRIBUTE TO DISEASE PATHOGENESIS OR ITS PROGRESSION

The “neurocentric” vision of mental disorders, which has prevalently dominated the research focus for many years, has hindered other approaches to study etiopathogenesis of neuropsychiatric disorders. Nevertheless, from the previously described functions of glia cell in the CNS, it has already become increasingly evident that an expansion of the focus from an exclusively “neurocentric” to an additionally “gliocentric” can strongly increase our understanding of the mechanisms underlying CNS disorders, thus improving our possibilities to better refine future therapeutic strategies.

Almost all epidemiologic and twin studies reported that several degrees of comorbidity can be found among neuropsychiatric disorders, hence suggesting that common mechanisms might be causative of disease etiopathogenesis and/or contribute to its progression. In the following paragraphs, we will highlight evidences that indicate how glia cells may be seen as a common “thread line” as susceptible cells that, when dysfunctional, might contribute to the development or progression of neuropsychiatric disorders. 

### 3.1 Major Depressive Disorder (MDD)

Major Depressive Disorder is a devastating mental illness, affecting about 10% of the worldwide population, with an enormous economic burden [39]. Major core symptoms of this disease comprise sad mood, loss of interest and energy, cognitive impairment, insomnia and loss of appetite. The late onset of antidepressant efficacy (three to four weeks after beginning of medication) and low response rates with full remission (30-50% of patients) still constitute major drawbacks in antidepressant therapies. Second and third generation of antidepressants have improved side effects of pharmacological profiles, but they still require several weeks of treatment before symptoms ameliorate. Moreover, in the many cases in which patients do not respond to a first treatment, a second antidepressant has to be prescribed with the consequent additional suffering of a prolonged course of the disease and decreased success of remission rates [40]. Nevertheless, all research efforts have failed the promise of producing newer or faster drugs for the treatment of depression.

Several studies reported about the involvement of astrocytes in MDD [41, 42]. Moreover, we have recently published a comprehensive review in which we discussed evidences on how this glia cell type might be affected in MDD [43]. 

Interestingly, also a role for oligodendrocytes in this pathology may be hypothesized, as a recent work has indicated a decrease in expression of oligodendrocyte specific genes in human temporal lobes of depressive patients [44]. Moreover, work from our colleagues who searched for potential biomarker instrumental as either diagnostic or therapeutic predictive tools, suggested the possibility of a glial involvement in MDD [45]. They indeed found that, among others, the complement C3 and ApoE, that are respectively related to pruning of synapses through the complement cascade [35] or induction of synapses through cholesterol [46], were reduced in the cerebrospinal fluid (CSF) of depressed patients. Because the protein content of CSF might directly reflect functional alterations in the CNS, the results of such studies may potentially indicate an involvement of astrocytes or microglia cell in the pathology of MDD, as far as the identified proteins are primarily expressed in these cells [47, 48].

More hypothesis-free approaches such as linkage studies additionally offered the possibility to identify susceptible candidate genes for specific disorders, with the hope to discover new pharmacological targets. One of the them reported an association of SNPs in the locus coding for P2RX7, a member of the purinergic ligand-gated ion channels of the P2X family, with MDD [49]. This channel is located on astrocytes and microglia cells, thereby strongly suggesting a link between astrocytes and microglia with MDD. Other studies identified MDD associated genes as candidates that induce vulnerability to develop the disease or facilitate its progression [50, 51]. From most of these studies especially genes related to the serotonin, dopamine or norepinephrine systems have been often selected and further discussed, because of the first hypothesis about mode of action of antidepressants *via *increased monoamine availability in the synaptic cleft. Nevertheless, an accurate screening of lists of affected genes made available through such studies revealed that also genes related to immune responses or inflammation pathways might be affected in the brain of MDD patients. Among them, for example, the TNFalpha system has been repeatedly indicated as dysfunctional in MDD [50, 52]. Together with interleukins, the cytokine TNFalpha is released from astrocytes and microglia cells during neuroinflammative processes that affect the brain and may be causative for neuropsychiatric or neurological disorders [53]. We hypothesize that a disrupted activation of P2RX7 in microglia due to single nucleotide polymorphisms (SNPs) [49] might trigger a consequent imbalance in its downstream molecular effectors like TNFalpha [54], thereby inducing an hyper- or hypo activation of microglia cells. As far as these cells are crucial for shaping neuronal circuits [36], we may speculate that such disrupted microglia activation may consequently impact pruning/formation of proper synaptic contacts either during development or in adult life, leading to early or late onset of MDD, respectively.

### 3.2 Bipolar Disorder (BD)

Bipolar disorder has a lifetime prevalence rate of 1-3% [55] and can be further subdivided into type I and II. Though some core symptoms may be similar to MDD, it additionally encounters episodes of mania (bipolar I, BPI) or hypomania (bipolar II, BPII), characterized by excessive euphoria, increased energy, decreased need for sleep, increased sexual desire and grandiose thoughts [56]. The manic symptoms may become so severe to remind of hallucinations and delusions typical of schizophrenia. Therefore, some comparative studies evaluated the possibility that the two diseases might share similar genetic components [57]. Twin studies have revealed a high degree of heritability set around 80-90% [58], indicating how the genetic background may play a crucial role in the etiopathogenesis of BD [59]. Therefore, genetic studies may be more informative about BD etiopathogenesis. Many reports showed a severe loss of oligodendrocytes and myelin in patients with BD [60], suggesting that this disease might depend on affected genes coding for proteins fundamental for survival of oligodendrocytes or myelin formation or that some deficiencies in other genes might be relevant to induce a consequent oligodendrocyte pathology. Indeed, genes coding for NMDA receptors have been found altered in BD [57]. It has been shown that signals from neurons are activating differentiation programmes in OPCs that are crucial for a proper myelination of axons [30] and that glutamatergic inputs on NG2 cells are existing that regulate their proliferation and distribution in the barrel cortex [33]. Therefore, if these inputs are using NMDA receptor to activate downstream differentiation/myelination programmes, it maybe hypothesized that a lack of proper NMDA receptor functionality underlies consequent disruption of oligodendrocyte physiological properties and leads to BD. Only one report until now showed that conditional deletion of NR1 subunit of NMDA receptors on NG2 cells seems to be dispensable for a regulation of their proliferation and subsequent myelination processes [61]. Nevertheless, it remains to be evaluated whether other receptor subunits that were found to be associated with BD, as NR2A or NR2B, may play a role in those processes; or whether other time windows of NMDA receptor inactivation might be relevant for BD onset.

Similar to MDD, an association between SNPs in P2XR7 gene locus and BD has been found in patients [62]. It might be interesting to investigate whether differences in the expression of P2XR7 in one or the other cell type (i.e. astrocytes or microglia cells) might be helpful to develop unbiased tools to distinguish among subcategories of MDD either with or without comorbid BD.

### 3.3 Anxiety Disorders

Anxiety disorders are often characterized by a high degree of comorbidity with MDD, but different subtypes exist that differentiate from MDD because of disease-specific symptoms [63]. Six subtypes of anxiety disorders can be defined [64]: generalized anxiety disorder (GAD), panic disorder (PD), specific phobia, obsessive-compulsive disorder (OCD), post-traumatic stress disorder (PTSD) and agora- or socio-phobias. Anxiety is not a pathological response *per se*, but it is the natural response of an individual to life threatening stimuli. It becomes pathological when this response becomes disruptive and hence interferes with the capabilities of an individual during the everyday life. Anxiety disorders are mostly characterized by different symptoms, depending on the specific subtype. Though their high comorbidity with MDD, several studies have pointed out on specific characteristics of anxiety disorders that distinguish them from MDD. For a general description of the various anxiety disorders, we might refer to an excellent still very actual book on the topic [65].

One common feature that can be found across different anxiety disorders is a lack of correct processing of fear stimuli, either as an excessive fear conditioning or as impaired fear extinction [66, 67]. It has been shown how these processes correlate to a modulation of synaptic contacts and we might therefore hypothesize that fear processing is dependent on glia cells [68].

A recent report by Ayling and colleagues [69] on the use of Diffusion Tensor Imaging (DTI) in anxiety disorders revealed that white matter is especially affected across anxiety disorders, suggesting this disruption of white matter as one common feature for all anxiety disorders, independent from their categorization. The white matter contains both fibre tracts, surrounded by myelin sheaths, and fibrous astrocytes, whose functions in the brain are not yet characterized. These findings indicate that a disturbed myelination or astrocytic functions are typical in anxiety disorders. It has been recently published that the ERK/MAPK signalling in oligodendrocytes is controlling thickness of myelin sheaths [70]. Therefore, it may be suggested that the morphological differences observed in the white matter may derive from a disrupted myelin formation, whose regulation is dependent on the ERK signalling. We will discuss later that indeed this signalling pathway has been indicated as a target of antidepressants which are also used for the treatment of anxiety disorders.

### 3.4 Schizophrenia (SCZ)

Symptoms of SCZ include positive symptoms such as delusions, disordered thoughts and hallucinations, typically regarded as manifestations of psychosis, and negative symptoms such as deficits of normal emotional responses or of other thought processes. While positive symptoms respond better to therapeutic interventions, typically with antipsychotics, negative symptoms do not respond that well. Linkage studies showed, for example, an association for NRG/ERB signalling with SCZ, with animal models indicating that this pathway is disrupted in SCZ [57]. An interaction between neuregulins (NRGs) and erB receptors has been described to be crucial for the proper differentiation of oligodendrocytes and the activation of their myelination programme [71]. Indeed, an extensive literature published over the last ten years revealed how several genes expressed specifically by oligodendrocytes are susceptible for SCZ [72]. In particular, protein products of such genes that are fundamental for proper development of myelin sheaths around axons to regulate their functionality have been found to be aberrant in schizophrenic patients [73]. To cite another example, a recent work characterized the proteomic profile of cerebella from transgenic mice for G72, a locus associated with genetic risk to develop SCZ, and found expression level differences in proteins related to myelination [74]. As far as other excellent reviews described far better the role of oligodendrocytes or OPCs in development or progression of SCZ [75-77], here we will mention only few examples on how some functions/dysfunctions of these cells might play a role in this disease. For more specific details, we may suggest the reader to refer to the aforementioned comprehensive reviews.

A molecule found to be disrupted in SCZ was named after this finding as Disrupted-In-Schizophrenia 1, DISC1 [78]. The protein product of DISC1 was shown to specifically interact with fasciculation and elongation protein zeta-1, FEZ1, an important regulatory process that direct fasciculation of axons and, when disrupted, may cause neurological disorders and altered development of cortical architecture [79, 80]. 

Current treatments for schizophrenic patients include antipsychotics, while drugs that block glutamatergic transmission like riluzole or ketamine can mimic some of the symptoms in animal models, suggesting that a lack of proper glutamatergic transmission may represent an important biological substrate in the pathogenesis of SCZ.

Indeed, it has been shown that genes coding for NMDA receptor subunits are affected in SCZ, which may account for a reduced glutamate signalling and may lead to SCZ symptoms [57]. In addition to that, drugs that modulate glutamate signalling, but acting on the functional regulation of intact NMDA receptor, have been shown to mimic SCZ symptoms. NMDA receptor function is actually controlled not only by direct binding of NMDA or glutamate, but also by the binding of two co-agonist, the amino acids glycine and D-serine [81]. A depletion of D-serine production, which is specifically occurring in astrocytes, in an animal model bearing a mutated form of DISC1 in astrocytes showed SCZ like phenotype, thereby indicating how a mutation in astrocytes can still indirectly affect the onset of SCZ symptoms [82]. 

### 3.5 Attention Deficit Hyperactivity Disorder (ADHD)

Attention Deficit Hyperactivity Disorder (ADHD) is a developmental disorder characterized by a variety of symptoms, typically including inattention, hyperactivity and impulsivity [83]. A recent article showed how this disorder may relate to a deficit in glia integrity [84]. Specifically, the authors remarked the glial protein S100ß as a putative biomarker of a glial pathology in the disease and suggested an excess of its release as causative of a possible disruption of neuronal responsiveness in the brain of ADHD patients. 

Another report suggested a correlation between astrocytes and ADHD through the protein SynCAM1. Mice carrying a dominant negative SynCAM1 in astrocytes develop behavioural abnormalities similar to disease symptoms observed in human patients [85]. Moreover, DTI studies demonstrated a disruption in frontocortical connectivity due to defects in white matter tracts [86]. Thus, indicative of defects in cells contained in the white matter, as we already mentioned for anxiety disorders (paragraph 3.3).

### 3.6 Autism Spectrum Disorder (ASD)

Autism spectrum disorder (ASD) is also commonly seen as a neurodevelopmental disease with a high genetic component characterized by impairment in social and language skills, cognitive deficits and stereotypic behaviours. It is considered to be dependent from multiple genetic and non genetic components [87]. The susceptibility of several genetic loci that are controlling many aspects of brain development has been associated with its prevalence [88]. Intriguingly in respect to other psychiatric disorders, for ASD it has been described a dysfunction in interneurons underlying its pathophysiology [89]. Several papers described morphological aberrancies in brains of ASD patients that include dysfunctions in neuronal growth affecting many areas which sustain cognitive functions (cortex) or motor coordination (cerebellum) [90]. Specifically, reports indicated a strong dependence of ASD on genes related to correct formation of synapses, which is also an underlying theme in epilepsy (see next paragraph), a syndrome frequently comorbid to ASD [91]. A misbalanced proportion of excitatory and inhibitory synapses has been observed, that may be dependent on defects in genes of the neuroligin/neurexin family, as far as the proper expression of neuroligins has important consequences on the correct development of a balanced inhibitory/excitatory neuro-transmission [92]. 

One interesting gene that had been long associated with autistic behaviours and has been extensively studied in animal models of ASD is *reelin* [93]. Interestingly, in addition to morphological defects due to disturbed neuronal migration dependent on a special type of glia cells, the radial glia [94] during development [95], the *reelin* product has been shown to act through one of its receptors, ApoE. We already mentioned this receptor when we discussed findings indicating an association of MDD with glia cell functions (paragraph 3.1). In brief, ApoE has proven its function in formation of excitatory synapses induced by cholesterol released from astrocytes [46]. It may be hypothesized that an interaction occurs among these signalling molecules, reelin and cholesterol *via *ApoE that might cooperate to regulate neuronal migration and subsequent differentiation with formation of proper amount of synapses, and is disrupted in ASD. Though it may sound very speculative, a similar “cooperation” between signalling pathways has been shown for a correct development of the cerebral cortex [96]. Furthermore, the release of reelin has been shown from oligodendrocytes, in addition to radial glia [97].

Unfortunately, due to the high variability of symptoms of ASD, there is a lack of reliable research findings about the effectiveness or safety of drug treatments for adolescents and adults with this disorder.

### 3.7 Epilepsy

Epilepsy is usually defined as a neurological disorder, because of the absence of cognitive symptoms typically associated with neuropsychiatric disorders. Nevertheless, some of its pathological features may be common to neuropsychiatric diseases and therefore deserve to be mentioned here. Moreover, pharmacological treatment of epilepsy includes the use of anticonvulsant drugs such as valproic acid, which proved their efficacy as first treatment or co-treatment (augmentative drugs) in some psychiatric diseases such as bipolar disorder or manic forms associated with MDD and anxiety diseases. Though epilepsy can not be defined as a single disorder, but more as a syndrome with several symptoms, a general common feature that characterizes it is an abnormal electrical activity in the brain associated with frequent seizures [98]. Yet not well described, a wrong synaptic pruning during postnatal developmental stages can lead to an exaggerated number of excitatory synaptic contacts that may induce seizures in animal models. A first report by Chu and colleagues [99] showed that mice with a deletion of the C1q protein failed to properly shape excitatory synaptic contacts in layer V of the cortex, thereby producing an epileptic phenotype. We mentioned already before that a proper signalling of the complement cascade through the C1q protein released from astrocytes that activate the C3 protein and its receptor on microglia cells is determinant for the modulation of synaptic pruning [35], thus suggesting a clear involvement of glia cells in the etiopathogenesis of epilepsy. 

In addition to this, foci of epileptic seizures are found around gliomas, an additional indication that a deregulated activity of severely impaired function of glia cells may lead to associated, though secondary, epileptic disorder [100].

A direct role of astrocytes in epilepsy was also demonstrated by the observation that astrocytes contribute to elevated glutamate release in epileptic tissue [101].

## 4 EVIDENCES OF GLIA CELL RESPONSIVENESS TO PHARMACOLOGICAL TREATMENTS: A COMPLEMENTARY “GLIOCENTRIC” APPROACH WITH HIGH THERAPEUTIC POTENTIAL

The “neurocentric” view of CNS diseases has additionally profoundly influenced orientation of research efforts into a deeper understanding of neuronal responsiveness to commonly prescribed pharmacological treatments. This view has for many years strongly prevented to take into consideration how glia cells might also respond to psychotropic drugs and how their functions may be modulated and consequently impact the surrounding neuronal environment. Moreover, the effectiveness of some treatments to resolve symptoms for different diseases also strongly suggests that cellular targets of medicaments to induce release or amelioration of clinical symptoms might be common across diseases. Recently, at least one other review discussed the theme of myelination as a central mechanism downstream to several psychotropic drugs [102].

An important difference that might pave the way to investigate novel potential pharmacological targets may arise from comparative studies that evaluate shared versus different mechanisms used by neuronal and non-neuronal cells to respond to current pharmacological treatments. Some research in this direction has been already performed and revealed important information regarding novel pathways involved in response to antidepressants, for example, in neurons or astrocytes [103]. Moreover, even the analysis of responses of the same cell type to drugs belonging to different therapeutic classes may offer new perspectives to identify selective molecules targeted by one but not the other pharmacological compound. Using this approach, we have indeed shed light on a crucial difference in the response of an astrocyte-like cell line, the C6 glioma cells, to antidepressants or antipsychotics, identifying ERK1 as a determinant downstream player that induces an ERK-dependent increased release of glial cell-derived neurotrophic factor (GDNF) after antidepressant treatment, but not with antipsychotics [104]. 

In addition to these approaches, the identification of “susceptible periods” of time during development or adulthood in which neurobiological or environmental factors may occur that can be correlated with the emergence of symptoms for these different disorders may greatly help to refine treatments. That is, a better understanding on how medical treatments might modulate the neuron-glia interface during these “sensitive” periods of time may be used to block or reverse pathological processes when they begin. 

### 4.1 Antidepressants

Recently, we published a comprehensive review on the role of astrocytes as direct targets of antidepressant (AD) therapy [43]. Therefore, here we will limit our summary to other cell types as potential targets of antidepressants therapy and how we might implement our knowledge for drug discovery. 

The ERK/MAPK signalling pathway has been largely indicated to be affected in MDD and to represent a common target of several ADs in astrocytes *in vitro* [105]. A recent report showed that this pathway is required for regulation of myelin thickness in oligodendrocytes, but is dispensable for OPC proliferation and oligodendrocyte differentiation [70]. These findings indicate that results on reduced ERK signalling obtained from human post-mortem brain tissues [106] might be indeed due to a reduced activity in either cell type, not only in neurons; but also that AD might use other cellular substrates in the brain for their efficacy in re-establishing homeostasis of neuronal circuits. This is a quite unexplored aspect regarding pharmacological treatments, mostly due to the first assumption that MDD derives from a reduced monoamine signalling in the brain. Nevertheless, it may be of primary importance to better understand how signalling pathways are modulated from ADs in each cell type, but also how each of these pathways might be differently affected by drugs in the various cell populations. 

As an example, the cytokine TNFalpha has been proven to be a molecular target of different ADs (fluoxetine, sertraline, paroxetine, fluvoxamine, citalopram and venlafaxine) in microglia cells after induction of an inflammatory response with lypopolysaccharide (LPS) [52, 107]. Another study reported a regulatory loop on glutamatergic signalling mediated by activation of P2XR7 on microglia cells triggering release of TNFalpha [108]. Thus, further supporting findings about P2XR7 as susceptible gene in MDD [49] possibly through its lack of activation on microglia cells to induce TNFalpha release, what may disrupt the glutamatergic regulatory loop and leads to the onset of MDD. 

### 4.2 Antipsychotics

In one of our studies, we observed that the antipsychotics quetiapine, clozapine and haloperidol would specifically regulate activation of only ERK2 in rat C6 glioma cells, a cell line used as model for astrocytes, when compared to the antidepressant reboxetine, which was instead activating both ERK isoforms ERK1 and ERK2 [104]. Recently, it has been shown that the conditional deletion of *Erk2* in the mouse cortex leads to a slowed maturation of oligodendrocytes already evident at P10 as measured with a delay in the expression of the myelin protein MBP in the corpus callosum, that was restored by P21 [109]. Though our analysis was done in astrocyte-like cells, it might well be that similar results can be obtained in oligodendrocytes. Thus, it may be of high importance to explore this eventuality to gain indications on whether oligodendrocytes are capable of cell-autonomous responses to antipsychotics and whether ERK2 were their favourite targeted molecular effector. Especially the delayed maturation of oligodendrocytes during an early postnatal phase in the mouse that corresponds to pre-adolescence stages in humans, suggests that functional consequences might occur from a hypoactive *Erk2* gene during this period of time. Moreover, a hypofunctional *Erk2* gene might induce diseases with early age onset, such as SCZ or ASD, that indeed require antipsychotics as first line medication. Yet maybe too speculative, such hypothesis can be confirmed or ruled out only with experimental evidences. At least one recent report associates already Erk2 with neuropsychiatric diseases, as far as mutant mice lacking *Erk2* shows a reduced development of the cerebral cortex, enhanced differentiation of progenitor cells into astrocytes with a resulting misbalanced proportion of astrocytes and neurons and behavioural deficits in associative learning. More importantly, the article reports that children with a chromosomal mutation in the region containing the locus for *Erk2* exhibit cognitive impairment and delayed development, all reminders of ASD symptomatology [110].

A study by Kimoto and colleagues [111] also revealed a direct effect of another antipsychotic of second generation (or “atypical antipsychotics”), olanzapine, on proliferation of oligodendrocytes. Specifically, they used an *in vitro* culture of OPC treated with the typical antipsychotic haloperidol and the atypical olanzapine, thereby demonstrating how both compounds could increase the number of viable OPCs, suggesting a protective role, but with a better efficacy for olanzapine. Moreover, their differentiation was inhibited, suggesting that the effects of antipsychotics might be mediated by the direct modulation of oligodendrocyte lineage.

### 4.3 Mood Stabilizers

This group of drugs is commonly used as primary treatment for the care of BD and to treat mania comorbid with other psychiatric disorders. In several cases, they are used as augmentative drugs in MDD as they can ameliorate some mood oscillations prevailing during the first weeks of treatment with antidepressants. Among them, lithium is the mostly renowned and also widely used. It is, nevertheless, very low specific, known to target several signalling pathways and to induce activation of many genes [112]. Nonetheless, a recent study demonstrated that lithium-mediated inhibition of its “classic” known target GSK3ß stimulates OPCs proliferation, oligodendrocytes differentiation and promotes myelination [113]. Indeed, this article sustains that the activity of lithium as mood stabilizer might help restoring neuronal circuits through a reactivation of the myelination programme, that has been found impaired in diseases such as BD or SCZ [60]. In line with these findings, a further article evidenced the power of lithium in remyelination, thereby underlying its potential in the treatment of neurodegenerative disorders [114]. It would be interesting to evaluate whether these results may be suitable to identify cell-type specific molecular targets of lithium that would represent good candidates for the development of higher selective drugs. 

Interestingly, it was shown in human astrocyte-derived cells (U-87 MG) that all four mostly used mood stabilizers, namely lithium (LiCl), valproic acid (VPA), lamotrigine (LTG) and carbamazepine (CBZ) can activate FEZ1 as common downstream target [115]. Functions of this gene has been previously characterized through identification of its main interaction partners, thus defining its biological functions mainly in neurons [116]. The finding of Yu and colleagues [115], therefore, opens important questions on novel functions that FEZ1 activation in astrocyte may imply. Among interaction partners, F-actin is an important factor to regulate astrocyte motility that is a critical feature of astrocytes to get in contact with other brain structures. Therefore, it would be of valuable information to know whether the activation of FEZ1 in astrocytes mediated by mood stabilizers may have consequent indirect effects on modulation of either structural plasticity or neuronal connectivity, which are impaired in BD and SCZ. 

A recent report from Perisic and colleagues [117] explored the activity of several antipsychotics and antidepressants on the epigenetic regulation of GLT-1 promoter in cultured rat primary astrocytes. GLT-1, a specific astrocytic glutamate transporter, plays an important role in the modulation of glutamate signalling [118]. Comparing the effects of the mood stabilizers VPA, LTG and CBZ with those of the antidepressants amytriptyline (AMI), venlafaxine (VEN) and citalopram (CIT), the authors showed that only VPA was able to induce global histone hyperacetylation. In addition, the comparison with antidepressants with respect to DNA methylation unmasked a specific, different efficacy of VPA in triggering additional effects on DNA methylation patterns, while AMI could only influence DNA methylation but not histone modifications. It would be of major interest to evaluate how such changes in epigenetic patterns between antipsychotics and antidepressants on other genes relevant for their respective targeted disorders may account for cellular specificity that might be used for refinement of therapeutic interventions. 

### 4.4 Anticonvulsants/Antiepileptics

Under the category “anticonvulsants” mood stabilizers may also be mentioned that we already discussed in the previous paragraph and benzodiazepines that will be talked about in a further paragraph (4.5). Here we will mention some additional drugs that we think that might be relevant for our aim to show glia responsiveness to pharmacological treatments. Gabapentin is a commonly used drug in the treatment of seizures. A paper by Eroglu and colleagues [119] elegantly showed how gabapentin receptor alpha2delta-1 represents the neuronal target of thrompospondins to trigger formation of excitatory synapses in the CNS. Gabapentin antagonizes the activation of the alpha2delta-1 receptor mediated by thrombospondins, thereby inhibiting excitatory synapse formation. Given the fact that thrompospondins released from astrocytes promote synapto-genesis [120] and an excess of excitatory synapses may be causative of seizures [99], it may well be hypothesized that the pharmacological efficacy of gabapentin might be based on a counteracting activity on astrocyte-mediated induction of synaptogenesis.

Pregabalin is an anticonvulsant currently used for treatment of fibromyalgia and neuropathic pain. Recent work revealed its properties to be ideal for the treatment of anxiety disorders, with faster onset of action in comparison to antidepressants and less withdrawal symptoms after treatment discontinuation than benzodiazepines [121]. Recent work showed that pregabalin can directly act on astrocytes *in vitro* and regulate glutamate transport [122]. Moreover, *in vivo* work indicated astrocytes as targets of the anticonvulsants valproate, gabapentin and phenytoin with a consequent decrease of Ca2+ activation responsible for an excessive release of glutamate in the epileptic tissue [101]. Other work *in vitro* showed that astrocytes are actually a direct target for several other antiepileptic drugs [123]. 

Unfortunately, much less is known about responsiveness of other glia cells to these drugs and work in this direction is recommended to gain a better overall picture and to improve treatments.

### 4.5 Psychostimulants

Typically, this class of drugs is used for the treatment of ADHD and in few cases in comorbid ASD. Methylphenidate is one of the most prescribed drugs for the treatment of ADHD. Only few report exist that study the effects of these drugs on glia cells. Among them, one very actual report showed that methylphenidate may cause activation of microglia cells in the substantia nigra of mice exposed to clinically relevant dosage of the drug [124]. Moreover, drug administration induced long term changes in brain circuits with major impact on both neuronal and glia markers. Especially changes in astrocytic regulation of glutamate uptake trigger consequences on synaptic rearrangements that influence behavioural responses [125]. Additionally, prolonged treatment of rats induces hypertrophic astrocytes, indicating that it may act directly on these cells [126].

Also for the second compound used as medication for ADHD, atomoxetin, a norepinephrine reuptake inhibitor (NRI), it has been shown how it may act on astrocytes [127]. In one of our studies we showed that another NRI, reboxetine, that is used as antidepressant, would act on an astrocyte-like cell line, the C6 cells, to activate ERK1 and ERK2 with consequent increase in release of GDNF [104]. It might be of interest to examine whether GDNF may additionally be activated by atomoxetin in astrocytes and with which physiological consequences.

### 4.5 Benzodiazepines

Benzodiazepines are commonly used as affective medication for anxiety disorders and comorbid psychiatric conditions or to treat seizures because of their fast onset of action. They act *via *direct binding to GABA_A_ receptors at a different benzodiazepine site that varies with GABA_A_ receptors´s subtype composition. Nevertheless, the development of withdrawal symptoms after treatment discontinuation and the cognitive and motor impairment call for a search of better drugs that are less susceptible to induce such liabilities [128]. Not much is known about the effects that benzodiazepines may exert on glia cells. One report described the expression of a benzodiazepine receptor, the peripheral benzodiazepine receptor (PBR), in microglia cells and how its activation after administration of benzodiazepines induces microglia cells proliferation and release of nitric oxide (NO) and TNFalpha. On the contrary, when cells had been previously activated with lypopolysaccharide to stimulate an inflammatory reaction *in vitro*, benzodiazepines would reduce release of both factors, NO and TNFalpha, suggesting a protective role of these drugs [129]. We described before how antidepressants are also targeting microglia cells to modulate release of TNFalpha. Because benzodiazepines are often used as additional therapy to antidepressants, the results presented suggest that these drugs might be additive, with benzodiazepines as fast onset agents that start protective effects before antidepressants become fully active. Another alternative hypothesis, however, comes from an interesting report that describes endozepines as endogenous ligands of benzodiazepine receptor. These molecules would be physiologically released by astrocytes [130]. Due to the reduced number of glia cells reported for several psychiatric disorders, we can postulate a consequent lack of endozepines in the brain that might cause a deregulation in GABAergic signalling. The reduced activity of the inhibitory system would then account for an imbalanced synaptic communication and excessive glutamatergic activity, which are typical hallmarks of, for example, depression and anxiety disorders. Benzodiazepines may then act in restoring homeostasis between these two major signalling systems in the brain.

Very recently, it has been better described the function of PBR, also called translocator protein (18kDa) (TSPO) that, upon ligand-mediated activation, induces proliferation of microglia cells and phagocytic processes [131]. Moreover, a study from our lab has indicated TSPO as a novel target for the development of anxiolytic without negative side effects of benzodiazepines [132]. Work in this direction may be highly relevant to experimentally evaluate how TSPO ligands may activate microglia cells and with which physiological consequences.

### 4.6 Drugs Targeting the Glutamatergic System 

The most well known drugs targeting the glutamatergic system can be found in a recent review by Mathew and colleagues [133]. They recently got a higher bulk of attention as it was shown that they can induce faster antidepressant effect in drug-resistant depressive patients [134]. Several reports showed first mechanisms of action of ketamine in animal models of depression to investigate biological substrates that induce such fast onset of action as an antidepressant [135, 136]. These studies identified mTOR as the signalling pathway activated as substrate of ketamine´s mode of action to induce synaptogenesis. Nevertheless, because glia cells are much active in regulating structural synaptic changes, it would be interesting to investigate whether these effects come as a consequence of direct neuronal or indirect glia cell responsiveness to ketamine. However, it has been reported that mTOR and ERK/MAPK signalling are both important pathways for the proper differentiation of oligodendrocytes [137], thereby suggesting that ketamine might also target these pathways in oligodendrocytes to exert its effects. Though the clinical use of ketamine as antidepressant still remains questionable because of its strong analgesic properties and the risk of abuse as recreational drug, these findings are encouraging research efforts to search for faster onset antidepressant drugs.

Riluzole is a glutamate-regulating drug mostly used in the treatment of amyotrophic lateral sclerosis (ALS). Recent advances on the function of astrocytes in glutamate metabolism prompted to explore the efficacy of this drug as antidepressant. Indeed, the results supported that riluzole might reverse depressive-like phenotype in rats *via *restoration of glial homeostasis [138].

It might be intriguing to evaluate whether potential differences in the regulation of D-serine production in astrocytes and/or its release to modulate NMDA receptors could account for the opposite effects observed for riluzole and ketamine as antidepressant or pro-schizophrenic drugs. One might expect to see opposite effects of these drugs on the modulation of D-serine, which may also account for their consequential beneficial or detrimental effects.

Clinical trials with direct administration of D-serine have indeed already shown a high therapeutic potential to treat SCZ [139], thus paving the way for further studies that focus on D-serine to deeply understand its modes of production, release and regulation in general as a possible candidate to develop or refine therapeutic strategies.

## CONCLUDING REMARKS

A better understanding of different mechanisms that lead to neuropsychiatric diseases can enormously increase our chances to reach the goal of personalized medicine, once we can identify how these mechanisms may vary in different patients. The validation of “omics” results using a plethora of *in vivo* and *in vitro* studies is absolutely mandatory for the identification of new clinically valid potential drug targets. Pharmacogenetics studies, which have highly improved our basic knowledge about disease mechanisms that correlate with responses of patients to different pharmacological treatments, have offered enormous advances in basic research and proposed many new potential targets for drug discovery. Nevertheless, the lack of complementary studies to evaluate the validity of such targets in different cell types has hindered advances in the production of more specific and/or effective pharmacological treatments in neuropsychiatry. Ideally, we can imagine that studies aimed at characterizing an “epigenetic code” that may represent a “fingerprint” of each individual patient may be of high importance as a tool to refine specific personalized treatments. However, also for this scope, parallel studies are recommended that evaluate how such epigenetic changes might have beneficial or detrimental effects depending on the cell-types that encounter such modifications. 

In too many cases novel compounds tested by pharmaceutical companies have failed to pass clinical trials at phase II or III, mostly because of lack of improvement in efficacy with respect to previously used compounds for the same indication or for an absolute failure to achieve any clinically relevant beneficial effects or, in worst cases, because of additional induction of undesired side effects. 

Advances in our understanding of the basic etiopathogenesis of most of the diseases that take into account heterogeneity of cell populations in the CNS have poorly paralleled development of technologies aimed at identifying molecular determinants of those diseases. The sad main consequence of such dearth of innovative discoveries is a dramatic reduction in global investments of pharmaceutical companies in R&D towards new drugs for the treatment of neuropsychiatric disorders. Nonetheless, mental disorders still represent the largest burden for many developed and developing countries, with, for example, depression estimated to become the second major cause of death by the year 2020 after (and comorbid with) cardiovascular diseases [39].

With this review we hope to have highlighted some of the latest evidences that indicate how fundamental is the gain of a global understanding of mechanisms of action of currently used drugs that consider both neuronal and non-neuronal contributions to their efficacy. We have resumed major findings in Table **1**.

We are aware of the fact that with only one review we could not cover the enormous amount of literature available on glia biology, involvement of glia cells in the etiopatho-genesis of all neuropsychiatric disorders and how glia cells respond to currently available pharmacological treatments.

However, we wish to have awakened a strong interest and increased sensibilization to further screen the literature in search of additional valuable information on how glia cells interrelate among each other and with neurons to modulate various functions of the whole brain in health and disease, and compare this information with results from pharmacological studies. Thus, enhancing our probability to formulate novel hypothesis at the neuron-glia interface about functions of brain networks in healthy and pathologic conditions. From several reports cited above, it seems for example that dysfunctional oligodendrocytes are an underlying thread of disorders with a psychotic component and that a deep understanding of their biology in health and disease might unmask novel potential and more specific targets amenable to drug discovery. Moreover, it is clear that morphologic changes observed in several psychiatric conditions may find their biological correlate in a reduced number of glia cells, in addition to the neuronal counterpart. Therefore, a better knowledge of cellular mechanisms that control proliferation, differentiation and integration into brain circuits of glia cells, that might restore physiological functions of the whole CNS may also help to identify novel targets to test new compounds with therapeutic potential.

## Figures and Tables

**Fig. (1):**
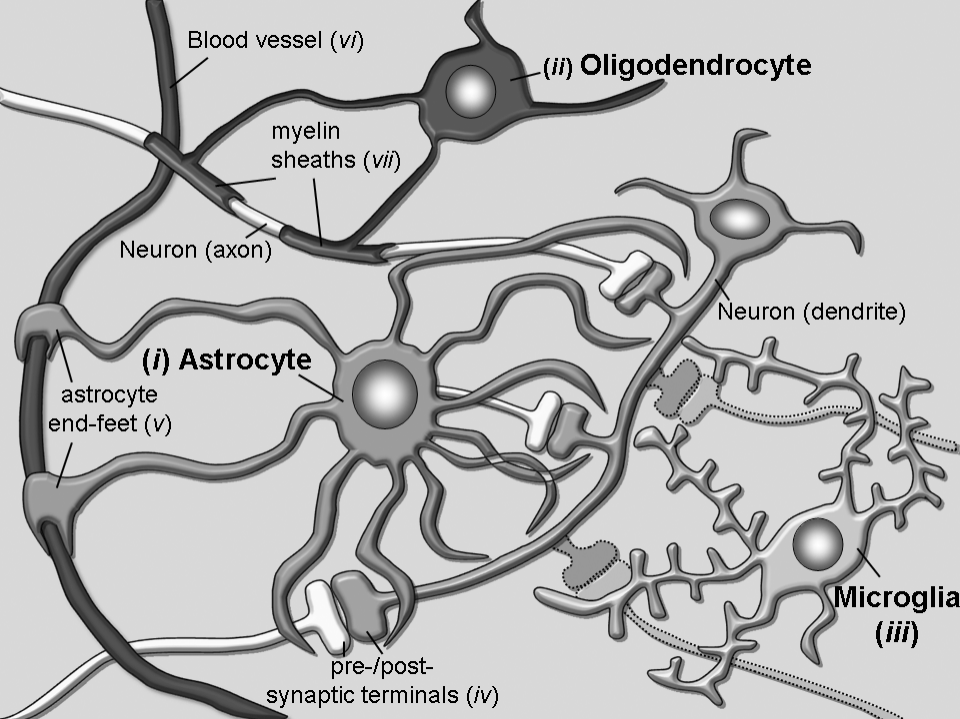
Several types of glia cells are populating the brain to sustain different functions. (*i*) Astrocytes contribute to regulation of neurotransmission through their processes wrapping around synapses (*iv*) and to modulation of the blood-brain-barrier and brain blood flow through their end-feet (*v*) surrounding blood vessels (*vi*). (*ii*) Oligodendrocytes are important to form the myelin sheaths (*vii*) around axons that guarantee conduction of electrical stimuli for long distances without increasing axonal diameters. (*iii*) Microglia cells represent the resident CNS immune cells and are fundamental surveyors of CNS extracellular environment that may help the maintenance or restore of homeostasis through pruning of inappropriate synaptic contacts.

**Table 1.** Neuropsychiatric Disorders are Due to Dysfunctional Glia Cells and Currently used Pharmacological Treatments Target Directly Glia Cells in Addition to Neurons

Neuropsychiatric Disorder	Glia Cells Affected (Direct/Indirect Evidences)	Pharmacotherapy	Glia Cells Responsiveness (Direct Effects)
Major Depressive Disorder	Astrocytes [14,36,104] Oligodendrocytes [5,39] Microglia cells [5,73]	Antidepressants Mood stabilizers	Astrocytes [36,38,59,100,132] Oligodendrocytes [11,62] Microglia cells [125]
Bipolar Disorder	Oligodendrocytes [27] Microglia cells [15]	Antipsychotics Mood stabilizers	Astrocytes [38,100,132] Oligodendrocytes [11,69]
Anxiety disorders	Astrocytes [10] Oligodendrocytes [10]	Antidepressants Antipsychotics Mood stabilizers Anticonvulsants/ Antiepileptics Benzodiazepines Drugs targeting glutamatergic system	Astrocytes [13,36,38,59,100,123,131,132] Oligodendrocytes [11,62,69] Microglia cells [31,125,128]
Schizophrenia	Astrocytes [74] Oligodendrocytes [16,24,45]	Antipsychotics	Astrocytes [38] Oligodendrocytes [69]
Attention Deficit Hyperactivity Disorder	Astrocyte [25,90,111] Oligodendrocytes [25]	Psychostimulants	Astrocytes [117] Microglia cells [129]
Autism Spectrum Disorder	Oligodendrocytes [115]	Antidepressants Antipsychotics Anticonvulsants/ Antiepileptics Psychostimulants	Astrocytes [36,38,59,100,117,123,131] Oligodendrocytes [62,69] Microglia cells [125,129]
Epilepsy	Astrocytes [123] Microglia cells [33]	Anticonvulsants/ Antiepileptics	Astrocytes [123,131]
